# Maternofetal Outcomes in Women With Congenital Uterine Anomalies

**DOI:** 10.7759/cureus.73430

**Published:** 2024-11-11

**Authors:** Shruti Ganti, Parimala Arogyaswamy, Jayashree Srinivasan, Priya Aarthy Archunan

**Affiliations:** 1 Department of Obstetrics and Gynaecology, Saveetha Medical College and Hospitals, Saveetha Institute of Medical and Technical Sciences, Saveetha University, Chennai, IND

**Keywords:** cesarean sections, congenital uterine anomalies, maternofetal outcomes, müllerian anomalies, obstetric complications

## Abstract

Müllerian anomalies are congenital disorders that affect the female reproductive system, often leading to a range of obstetric complications. These anomalies include structural abnormalities such as arcuate, septate, unicornuate, and bicornuate uteri, which can impact fertility, pregnancy outcomes, and delivery methods. This case series presents five patients with different types of Müllerian anomalies, including septate, arcuate, and unicornuate uteri. Each case is discussed concerning its presentation, diagnostic process, therapeutic interventions, and outcomes. These cases highlight the complications associated with these anomalies, including recurrent pregnancy loss, preterm delivery, and the necessity of cesarean sections. This series underscores the importance of early diagnosis and individualized management of Müllerian anomalies to improve obstetric outcomes. Advanced imaging techniques and surgical interventions, such as hysteroscopic metroplasty, can enhance reproductive success and reduce pregnancy-related complications.

## Introduction

Müllerian duct anomalies (MDAs) are congenital malformations resulting from incomplete or abnormal development of the paramesonephric (Müllerian) ducts during embryogenesis [1]. These anomalies can affect the uterus, cervix, and upper two-thirds of the vagina, leading to a wide spectrum of reproductive challenges [2]. The prevalence of Müllerian anomalies is estimated to be around 3% to 4% in the general population, but it can be as high as 25% in women with recurrent pregnancy loss or preterm delivery [3,4]. These anomalies are typically classified into categories such as septate, bicornuate, arcuate, unicornuate, and didelphys uteri, each with distinct reproductive implications. For example, a septate uterus, characterized by a fibrous or muscular septum dividing the uterine cavity, is strongly associated with reproductive failures, including miscarriages and preterm births [5,6].

Early and accurate diagnosis of Müllerian anomalies is crucial for improving reproductive outcomes. Historically, many of these anomalies went undiagnosed until complications arose during pregnancy or delivery. However, advances in imaging techniques, such as three-dimensional ultrasound and magnetic resonance imaging (MRI), have greatly enhanced the detection of these conditions, allowing for interventions before adverse outcomes occur [7,8]. Additionally, surgical interventions such as hysteroscopic metroplasty have been shown to be effective in restoring normal uterine anatomy in cases of septate uterus, thus reducing the risk of miscarriage and improving live birth rates [9,10].

Despite these advances, managing pregnancies complicated by Müllerian anomalies remains challenging due to the increased risks of preterm birth, malpresentation, and the need for cesarean delivery [11]. This case series aims to contribute to the existing literature by providing detailed accounts of five patients with different types of Müllerian anomalies, highlighting the diagnostic challenges, treatment strategies, and outcomes associated with each case. By examining these cases, we emphasize the importance of individualized care and the potential for positive outcomes with appropriate management [12].

Here, we present a case series of five women who were found to have uterine anomalies and provide a follow-up on their pregnancy outcomes and adverse events encountered at Saveetha Medical College between April and September 2024.

## Case presentation

Case 1

**Figure 1:**
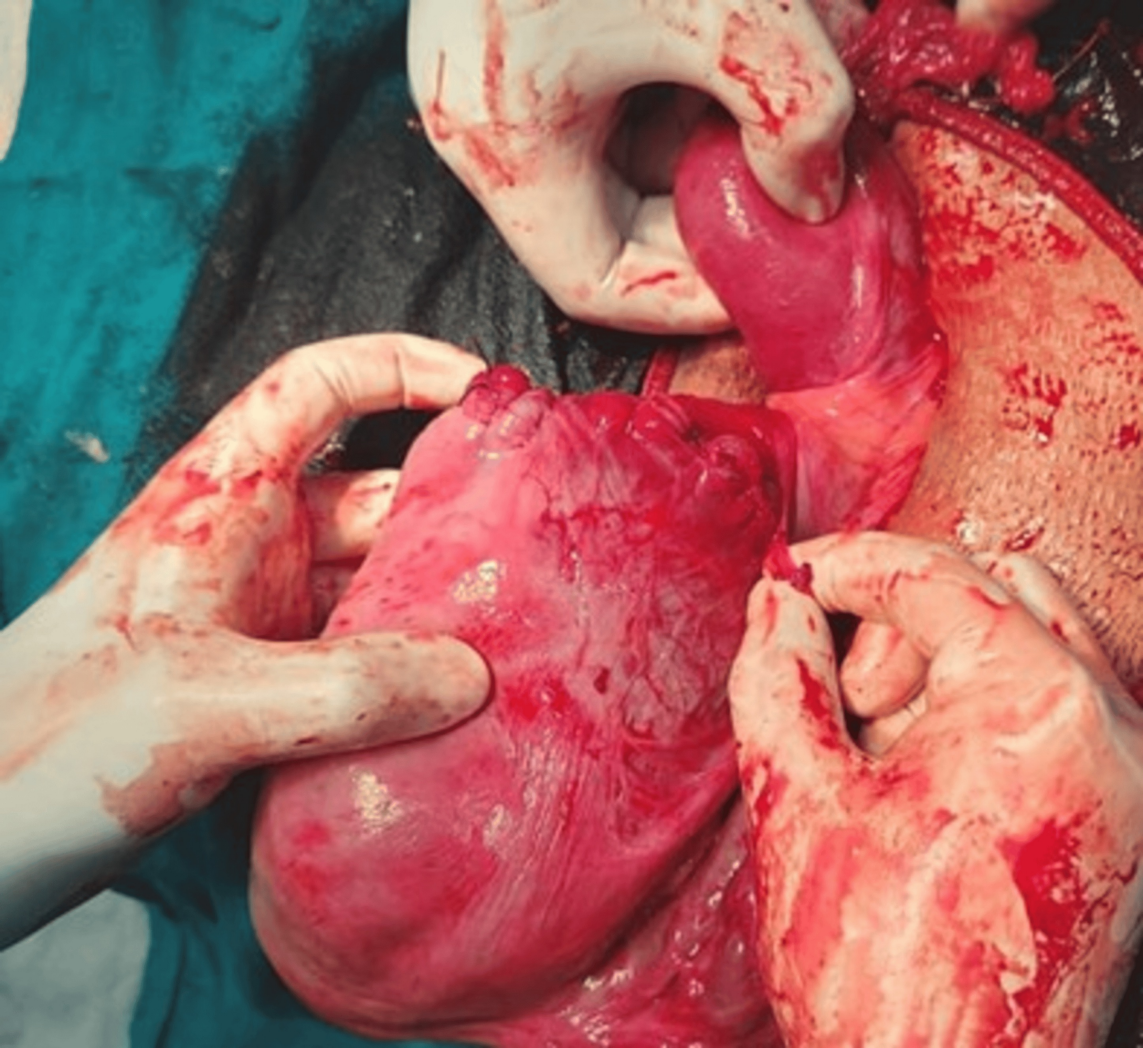
Bicornuate uterus with single live intrauterine gestation in the right horn with no communication.

A 30-year-old patient, gravida 3, alive 2, at 32 weeks and four days of gestation with a history of premature pre-labor rupture of membranes (PPROM), presented with complaints of leaking per vagina for one day. Her obstetric history included two spontaneous abortions at 14 and 16 weeks, for which dilation and curettage were performed. During her antenatal care, a bicornuate uterus with a slug in the right horn and no communication between the horns was diagnosed on the nuchal translucency (NT) scan and confirmed by MRI. She was regularly followed up and given steroid coverage. An emergency lower-segment cesarean section (LSCS) was performed due to preterm breech presentation and oligohydramnios. Intraoperatively, the right-side tube and ovary were visualized along with the uterine fundus, while the left side revealed another uterine fundus with a single tube and ovary, confirming a bicornuate uterus with no communication between the horns (Figure 1). The neonatal outcome was a preterm baby weighing 1.8 kg with APGAR scores of 7/10 and 8/10, requiring neonatal intensive care unit (NICU) care for 15 days. The maternal outcome included mild atonic postpartum hemorrhage (PPH) without excessive blood loss. The European Society of Human Reproduction and Embryology (ESHRE) classification for this case was U3cC0V0.

Case 2

**Figure 2:**
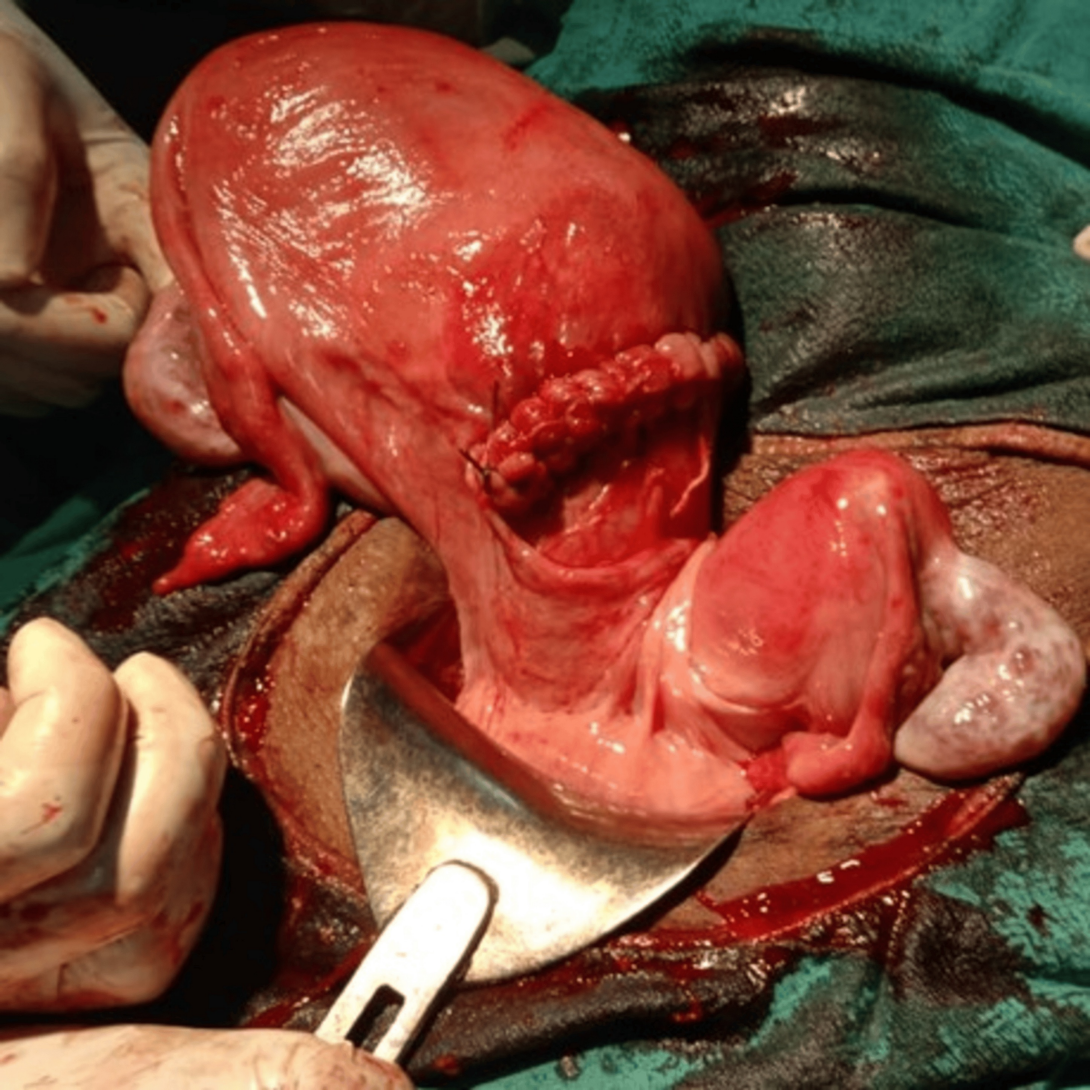
Bicornuate unicollis with single live intrauterine gestation in the right horn with communication.

A 26-year-old primigravida at 28 weeks and four days of gestation, with a bicornuate unicollis uterus and suspected small for gestational age fetus in the right horn following spontaneous conception, presented with complaints of leaking per vaginam. She was diagnosed with a bicornuate uterus at 12 weeks during the NT scan and was on regular follow-up. On examination, her abdomen showed a relaxed uterus consistent with 28 to 30 weeks, with the fetus in a cephalic position and a fetal heart rate of 140 beats/minute. A clear leak was noted on per speculum examination, and the patient was given a steroid and magnesium sulfate cover. An emergency LSCS was performed due to fetal distress with breech presentation. Intraoperatively, a breech extraction was done, revealing the right-side tube and ovary along with the uterine fundus, and the left side showed another uterine fundus with a single tube and ovary. There was communication between the two corpora at the lower level, confirming a bicornuate unicollis uterus with a single cervix (Figure 2). The neonatal outcome was an alive but extremely preterm baby boy, weighing 910 g, with APGAR scores of 4/10 and 8/10. The baby required prolonged NICU care but unfortunately succumbed to sepsis and acute respiratory distress syndrome. The maternal outcome was uneventful postoperatively. The ESHRE classification for this case was U3cC0V0.

Case 3

**Figure 3:**
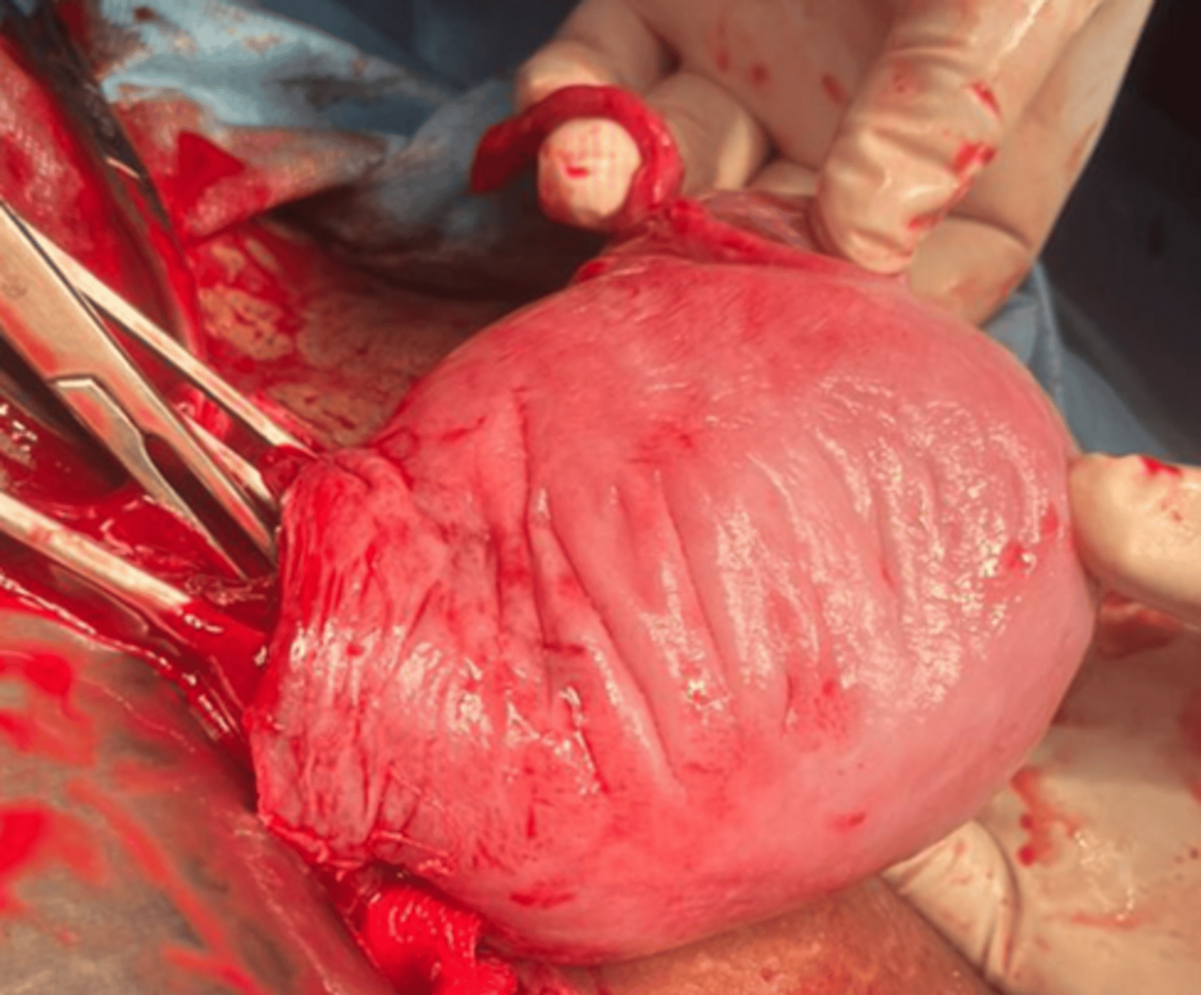
Unicornuate uterus with cephalic presentation.

A 19-year-old primigravida at 35 weeks and four days of gestation, following a spontaneous conception, presented with complaints of leaking per vaginam and abdominal pain. She was diagnosed with small for gestational age in the right horn during an NT scan, which was confirmed by MRI, and was regularly followed up with steroid cover given at 34 weeks. On examination, her abdomen revealed a relaxed uterus consistent with 34 weeks of gestation, with the fetus in a cephalic presentation, and a clear leak was noted on per speculum examination. An emergency LSCS was performed due to fetal distress. Intraoperatively, a live cephalic baby was delivered, and the uterus was inspected, revealing a unicornuate uterus with one fallopian tube and ovary present on the right side, both appearing normal. The left side had no fallopian tube, ovary, or rudimentary horn, confirming the diagnosis of a unicornuate uterus (Figure 3). The neonatal outcome was a live, late preterm boy with a birth weight of 2.21 kg and APGAR scores of 6/10 and 8/10, requiring NICU care. The maternal outcome was stable, with no excessive blood loss, and postoperative anemia was corrected with iron sucrose (hemoglobin 9.0 g/dL). The ESHRE classification for this case was U4C0V0.

Case 4

**Figure 4:**
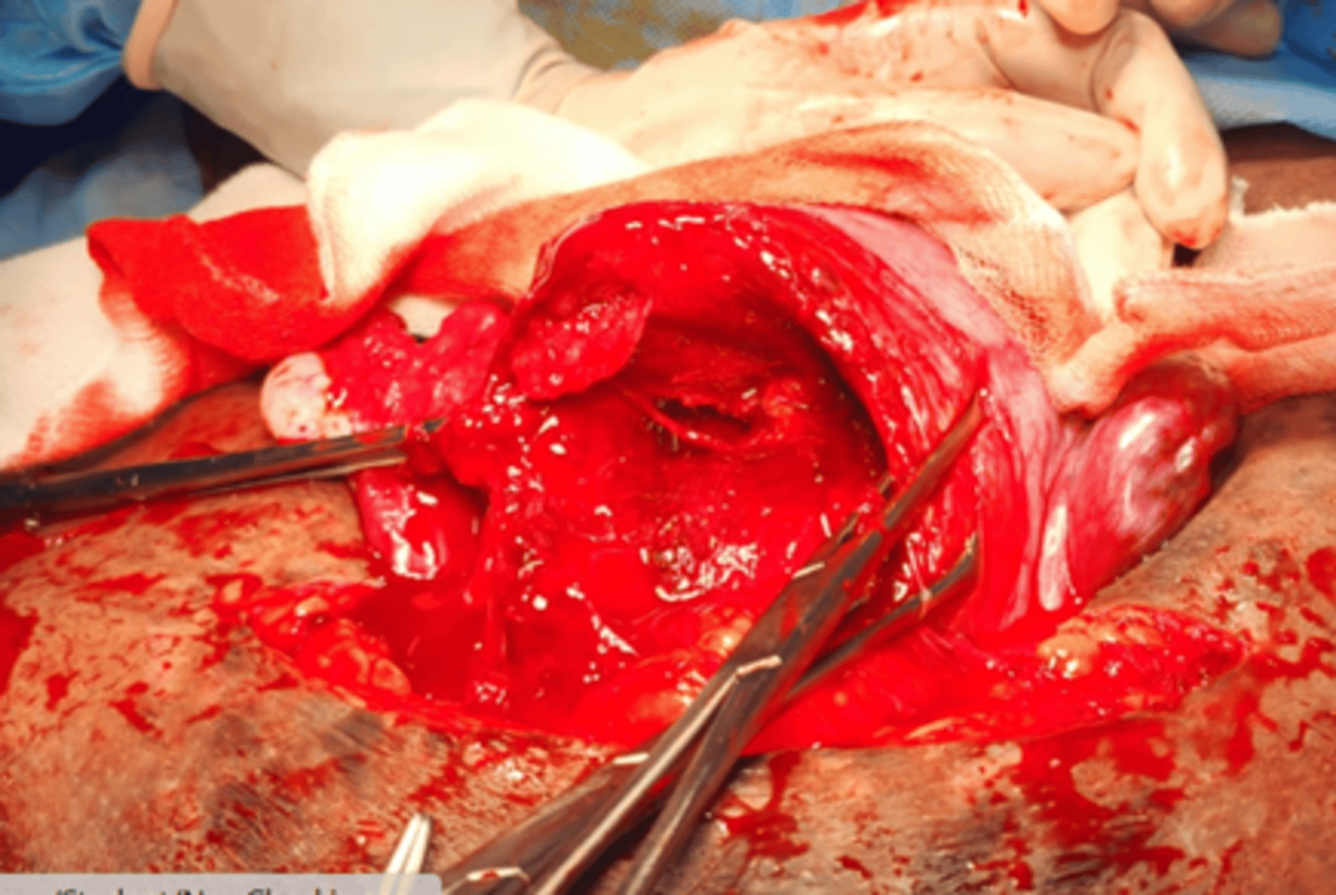
Complete septate uterus with single live intrauterine gestation in the left horn.

**Figure 5:**
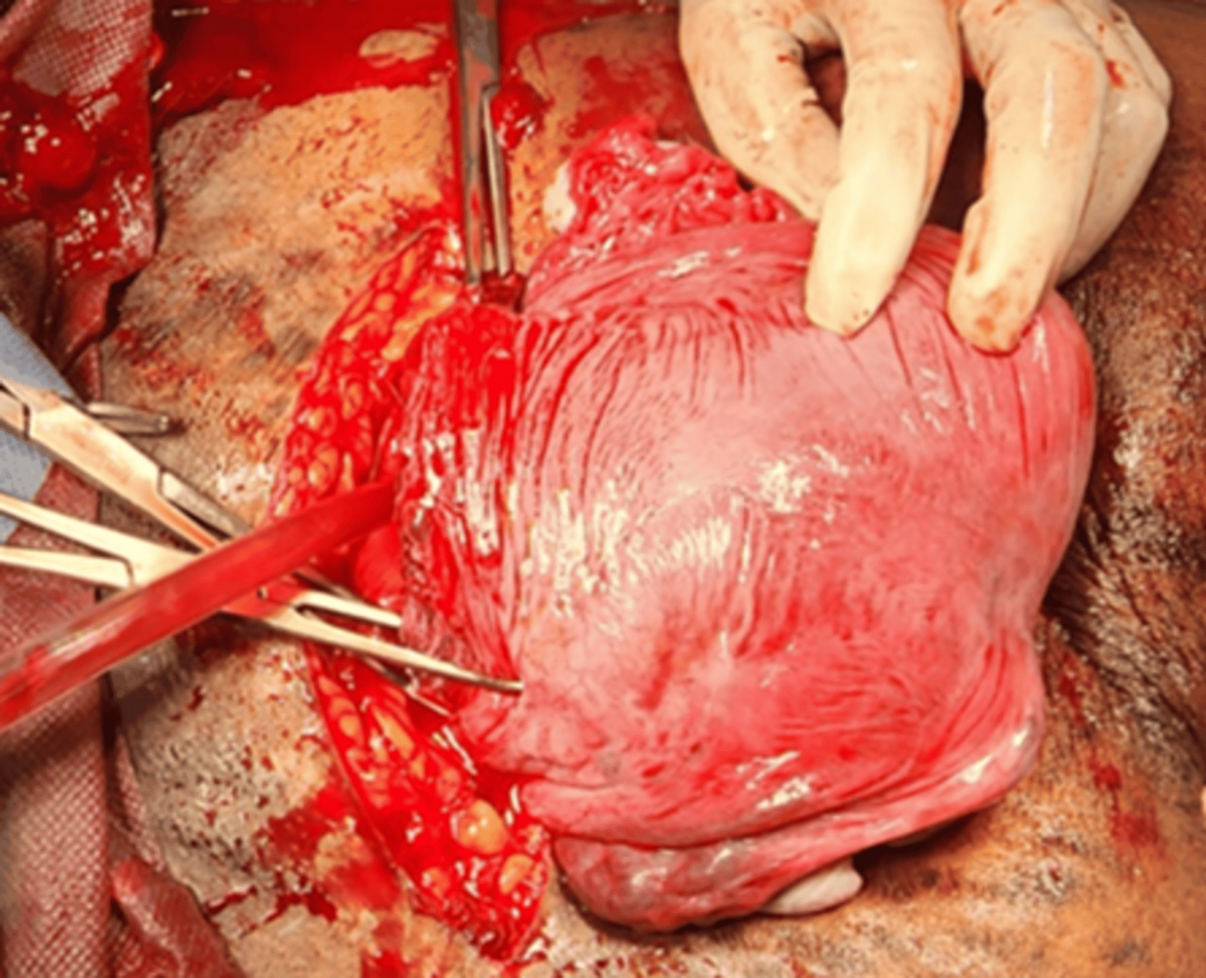
Complete septate uterus with single live intrauterine gestation in the left horn.

A 25-year-old, gravida 2, alive 1, patient at 33 weeks and three days of gestation, following a spontaneous conception, presented with complaints of intermittent abdominal pain for two days. She received steroid coverage before delivery. On examination, her abdomen revealed a uterus consistent with 34 weeks, with an irritable uterus and a fetal heart rate of 148 beats/minute. An emergency LSCS was performed due to a preterm breech presentation. Intraoperatively, a breech delivery was carried out, and a complete septate uterus was noted, with the septum extending into the uterine cavity. Both fallopian tubes and ovaries were normal (Figures 4, 5). The neonatal outcome was a live baby girl weighing 2.6 kg, with an APGAR score of 8/10. The maternal outcome included mild atonic PPH, which was medically managed, and the postoperative period was uneventful. The ESHRE classification for this case was U2BC0V0.

Case 5

**Figure 6:**
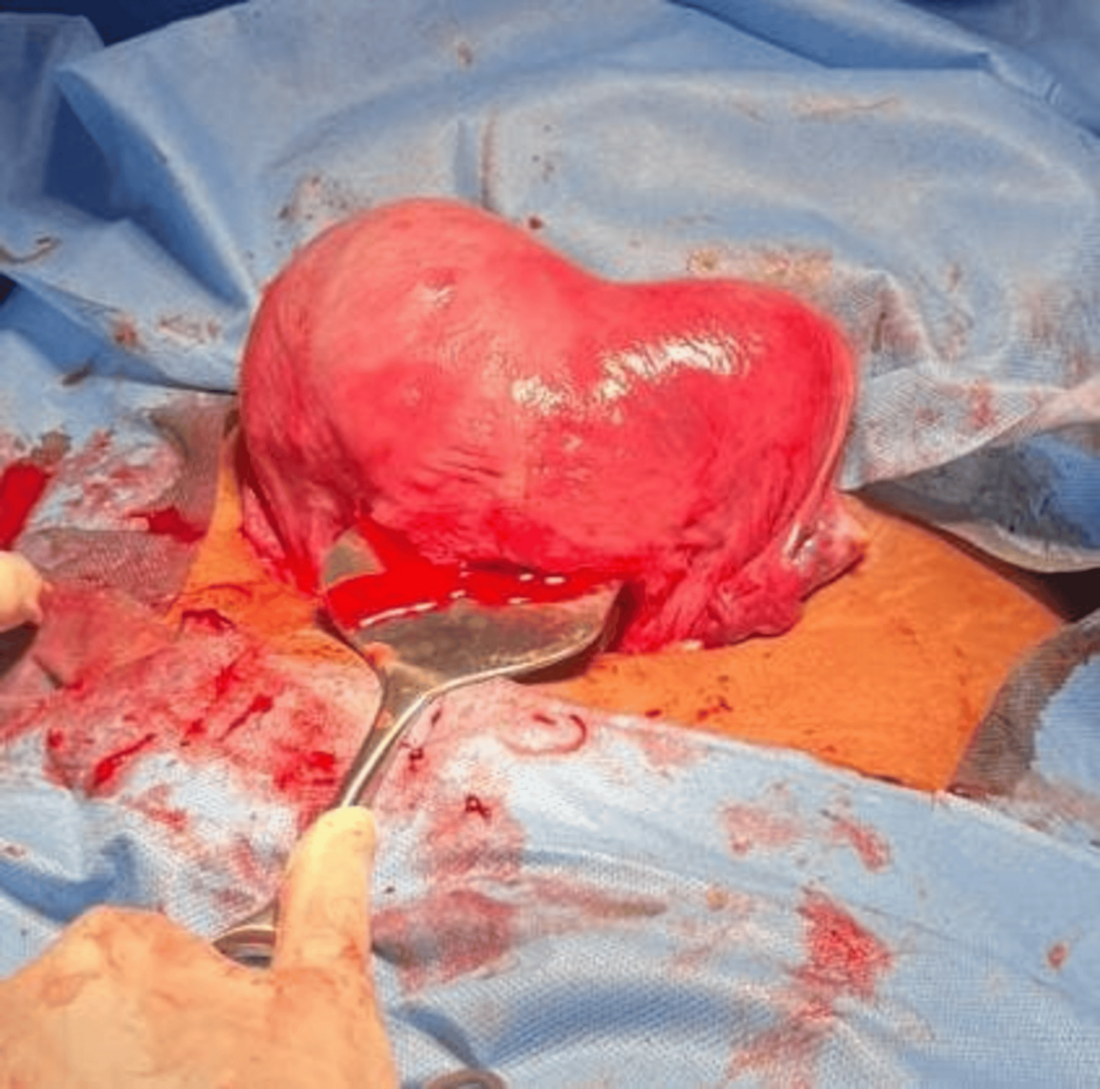
Partial septate uterus with malpresentation.

A 24-year-old, gravida 2, parity 1, live 1, patient with a history of a previous LSCS presented with complaints of pain over the previous suture site. Her past obstetric history included a cesarean section two years ago for a male baby weighing 2.7 kg due to breech presentation with premature rupture of membranes (PROM); the baby was born alive and healthy. On examination, the uterus was at term, with a breech presentation at the lower pole. A suprapubic transverse scar was present with tenderness, and the fetal heart rate was 140 beats/minute. The patient underwent an emergency LSCS with bilateral tubal sterilization due to a previous LSCS with scar tenderness and breech presentation. Intraoperatively, a partial septum measuring about 1 cm was noted, with two uterine cavities, while the bilateral ovaries and fallopian tubes appeared normal, confirming a diagnosis of a partial septate uterus (Figure 6). The neonatal outcome was a term baby girl weighing 3.33 kg, with APGAR scores of 8/10 and 9/10. The maternal outcome was stable, with no excessive blood loss or uterine atonicity, and the postoperative period was uneventful. The ESHRE classification for this case was U2AC0V0.

## Discussion

MDAs are congenital malformations resulting from aberrant development of the paramesonephric ducts during embryogenesis. These anomalies manifest in various forms, including septate, arcuate, unicornuate, and bicornuate uteri, and pose significant challenges in diagnosis, management, and pregnancy outcomes. These anomalies often remain asymptomatic until reproductive issues arise, making diagnosis difficult. Traditional diagnostic methods such as two-dimensional ultrasonography have limitations in accurately characterizing these anomalies. However, advancements in imaging, particularly three-dimensional ultrasonography and MRI, have significantly improved diagnostic accuracy [1,2].

These imaging techniques allow for better visualization of uterine morphology, aiding in the precise classification of anomalies. For instance, MRI can differentiate between a septate and bicornuate uterus, which is crucial for management decisions as the treatment strategies differ significantly [3]. In this case series, accurate diagnosis was paramount in guiding management decisions. The use of advanced imaging played a crucial role in identifying the specific type of MDA in each patient, allowing for appropriate therapeutic approaches. Routine advanced diagnostic imaging is essential for women with a history of infertility, recurrent pregnancy loss, or adverse pregnancy outcomes. MDAs are associated with a variety of adverse pregnancy outcomes, including recurrent miscarriage, preterm birth, malpresentation, and an increased rate of cesarean delivery. The severity of these outcomes often correlates with the type of anomaly. For example, patients with a septate uterus, the most common MDA, are at a significantly higher risk of early pregnancy loss due to impaired uterine cavity space and suboptimal endometrial environment [4].

Surgical intervention, such as hysteroscopic resection of the septum, has been shown to improve outcomes by increasing the likelihood of a successful full-term pregnancy [5]. In contrast, anomalies such as the unicornuate uterus, which results in a single functional uterine horn, pose different risks, including a higher incidence of preterm birth, intrauterine growth restriction, and malpresentation due to the reduced uterine space [7]. The cases in this series reflect these risks, with several patients requiring preterm cesarean sections due to complications associated with their uterine anomalies. The outcomes in these cases highlight the need for vigilant antenatal monitoring and a high index of suspicion for complications that may arise in the context of MDAs. The management of pregnancies complicated by MDAs is highly individualized, depending on the type and severity of the anomaly, as well as the patient’s obstetric history. Surgical correction, particularly for septate uteri, has been shown to significantly improve reproductive outcomes. Hysteroscopic metroplasty, a minimally invasive procedure, can correct the septum, reducing the risk of miscarriage and improving the chances of a successful pregnancy [5,8].

In this series, patients who underwent surgical correction had notably better outcomes, with successful term deliveries and fewer complications. However, not all anomalies are amenable to surgical correction. In cases such as unicornuate or bicornuate uterus, where surgical options are limited, management focuses on close monitoring throughout the pregnancy to manage and mitigate risks such as preterm labor and malpresentation [9]. The importance of a multidisciplinary approach cannot be overstated, involving obstetricians, radiologists, and sometimes surgeons to ensure optimal care. The prognosis for women with MDAs varies widely depending on the type of anomaly and the success of any corrective procedures. While advances in diagnostic imaging and surgical techniques have improved outcomes, the presence of a Müllerian anomaly still poses significant risks to both maternal and fetal health [11]. The cases in this series highlight the importance of early diagnosis and personalized treatment plans. For some women, particularly those with more severe anomalies such as unicornuate or didelphys uteri, the risk of adverse outcomes remains high despite optimal management.

The case series by Kohli et al. provides key insights that resonate strongly with the outcomes observed in our current cases, particularly concerning septate and unicornuate uteri. For instance, Kohli et al. reported a 23-year-old primigravida at 32 weeks with a complete septate uterus who experienced PPROM and severe oligohydramnios, leading to an emergency cesarean due to fetal distress. Similar to our findings in patients with septate uterine anomalies, this case underscores the association between septate uterus, preterm labor, and fetal malpresentation, ultimately necessitating NICU care for the newborn due to low birth weight and prematurity [2]. Another case by Kohli et al. involved a 31-year-old primigravida with a unicornuate uterus presenting at 35 weeks with severe oligohydramnios, growth restriction, and breech presentation, requiring NICU support post-cesarean section. This correlates with our findings in unicornuate uterus cases, emphasizing how reduced uterine space contributes to fetal malpresentation and complications such as preterm birth and growth restriction [2].

Similarly, Rao et al. described a 28-year-old multigravida with a bicornuate uterus at 36 weeks who presented with hand and cord prolapse, necessitating emergency cesarean section. This case echoes the risks associated with bicornuate uterus observed in our series, where structural malformations predispose patients to malpresentation and preterm labor [1]. Fox et al. detailed a case of a 30-year-old woman with a didelphys uterus who faced recurrent pregnancy losses, eventually requiring close monitoring and cesarean for preterm breech delivery, illustrating the impact of uterine anomalies on recurrent miscarriage and adverse pregnancy outcomes [3].

Raga et al. further reported that live birth rates were the highest in arcuate uterus cases (82.7%) compared to the lower live birth rates in bicornuate (62.5%) and septate (62%) uteri. This finding aligns with our observations that malpresentation and preterm labor, as well as the need for cesarean sections, were more pronounced in cases with bicornuate and septate uteri, mirroring the findings of Raga et al. [6]. Additionally, PROM was prevalent across our cases, supporting findings by Hua et al. that PROM rates are notably higher in patients with uterine anomalies. This connection between uterine anomalies and adverse outcomes, including the need for cesarean delivery due to malpresentation, reinforces existing literature and highlights the importance of tailored management for patients with MDAs [1,13].

The unicornuate uterus, the rarest of uterine anomalies, was represented by a single case in our study. This patient carried the pregnancy to term but delivered via cesarean section due to breech presentation. Supporting this, Ludmir et al. reported a high rate of pregnancy loss (80%) in cases of unicornuate uterus [14]. Patients with major fusion defects, such as unicornuate uterus, often have unilateral placental implantation, potentially leading to the exclusion of one uterine artery from uteroplacental circulation. This could explain the increased risk of outcomes such as birth weight below the fifth percentile and preeclampsia observed in our study, as these are likely related to uteroplacental insufficiency [12].

Future research should focus on refining diagnostic criteria and developing new surgical techniques that can offer better outcomes for women with non-septate anomalies. Additionally, long-term studies on the outcomes of pregnancies in women with MDAs, particularly after surgical intervention, are needed to provide clearer guidance on managing these complex cases.

## Conclusions

MDAs present significant challenges in reproductive health, particularly in terms of diagnosis, management, and pregnancy outcomes. This case series highlights the variability in presentation and outcomes, emphasizing the need for early diagnosis and individualized care. Surgical intervention, particularly in cases of septate uterus, can dramatically improve outcomes, but careful management is required for other types of anomalies to ensure the best possible results for both mother and child. The ongoing development of diagnostic and therapeutic approaches holds promise for improving the reproductive success of women with these challenging conditions.
